# An Uncommon Site for a Common Tumor: Pleomorphic Adenoma of the Accessory Parotid Gland

**DOI:** 10.7759/cureus.108170

**Published:** 2026-05-03

**Authors:** Praneeth V Tenneti, A Ravi, Leena Joseph

**Affiliations:** 1 General Surgery, Sri Ramachandra Institute of Higher Education and Research, Chennai, IND; 2 Pathology, Sri Ramachandra Institute of Higher Education and Research, Chennai, IND

**Keywords:** accessory parotid gland, benign salivary tumor, head and neck neoplasms, mid-cheek mass, parotidectomy, pleomorphic adenoma, rare salivary tumor, salivary gland neoplasms, stensen’s duct, surgical excision

## Abstract

Tumors of the accessory parotid gland are rare and often misdiagnosed because of their anterior location and proximity to the facial nerve. We report a case of pleomorphic adenoma arising from the accessory parotid gland in a middle-aged man presenting with a mid-cheek swelling for 20 years. Magnetic resonance imaging demonstrated a well-circumscribed lesion along Stensen’s duct. Complete surgical excision was achieved with preservation of facial nerve function. Awareness of this uncommon entity and appropriate surgical planning are essential to ensure optimal outcomes.

## Introduction

The accessory parotid gland (APG) is a distinct salivary tissue mass located anterior to the main parotid gland along Stensen’s duct [1]. It represents a normal anatomical variant and is present in a considerable proportion of the population, although it is infrequently recognized clinically due to its small size and deep location in the mid-cheek region [2]. Histologically, the APG resembles the main parotid gland and contains both serous and mucinous elements, with drainage occurring via accessory ductules into Stensen’s duct.

Tumors arising from the APG are rare, accounting for approximately 1-8% of all parotid neoplasms [3]. Notably, these tumors have been reported to exhibit a relatively higher rate of malignancy compared to those originating in the main parotid gland. Among benign lesions, pleomorphic adenoma is the most common histological subtype and typically presents as a slow-growing, painless swelling in the mid-cheek. Due to its location, it may be misdiagnosed as a lesion of the buccal space or subcutaneous tissue, posing a diagnostic challenge.

Imaging plays a key role in evaluation, with magnetic resonance imaging being particularly useful for precise localization and assessment of the tumour’s relationship to adjacent structures. Fine-needle aspiration cytology can assist in preoperative diagnosis, although its diagnostic accuracy may vary.

Owing to their atypical location and close relationship to the buccal branches of the facial nerve, APG tumors present unique surgical challenges [4]. Complete surgical excision with preservation of facial nerve function remains the treatment of choice. This report presents a case of pleomorphic adenoma of the accessory parotid gland and discusses its clinical presentation and surgical management.

## Case presentation

A middle-aged man presented with a painless swelling over the right side of the face that had been slowly increasing in size over 20 years. There were no associated symptoms such as facial weakness, pain, or salivary dysfunction.

**Figure 1:**
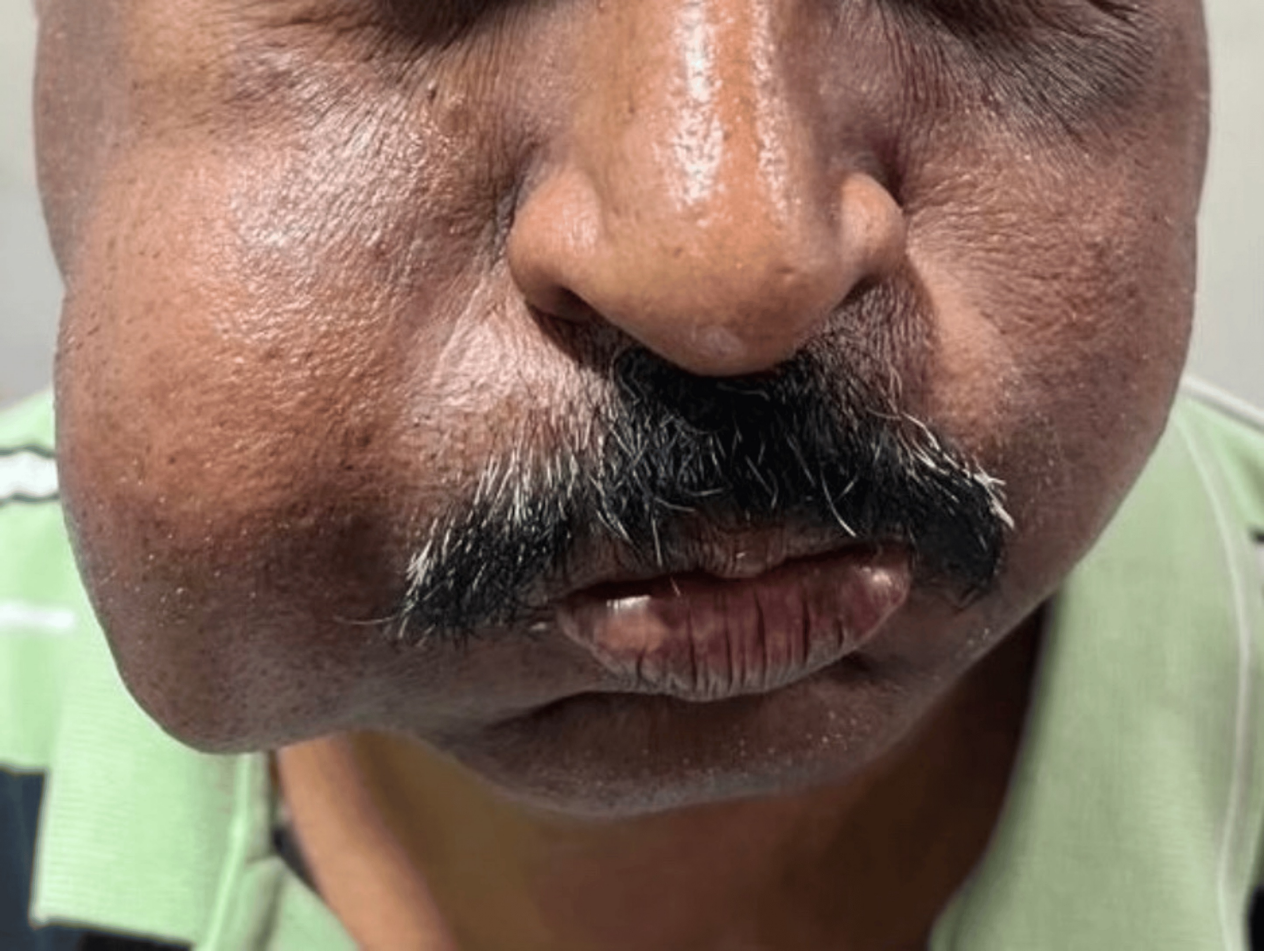
Preoperative image of the patient demonstrating a visible, well-defined swelling over the right mid-cheek region, causing mild facial asymmetry without overlying skin changes

On examination, a 4 × 4 cm, firm, mobile, non-tender mass was noted in the right mid-cheek region. The overlying skin was normal, and no cervical lymphadenopathy was detected. Examination of the contralateral parotid region was unremarkable (Figure 1).

Investigations

**Figure 2:**
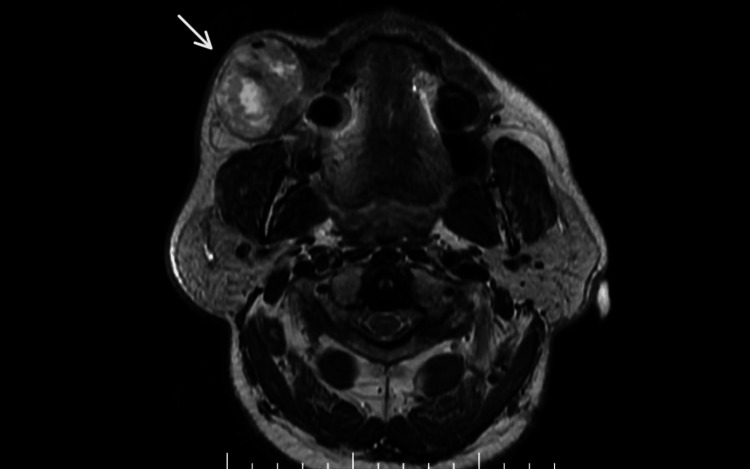
Magnetic resonance imaging showing a well-circumscribed, predominantly solid, heterointense lesion in the subcutaneous plane of right cheek The lesion was located along the course of Stensen’s duct, causing elevation and compression of the duct with proximal sialectasis. The white arrow indicates the location of the lesion.

Magnetic resonance imaging of the face revealed a well-circumscribed, predominantly solid, heterointense lesion in the subcutaneous plane of the right cheek. The lesion was located along the course of Stensen’s duct, causing elevation and compression of the duct with proximal sialectasis. These findings were suggestive of a benign tumor arising from the accessory parotid gland (Figure 2).

Treatment

**Figure 3:**
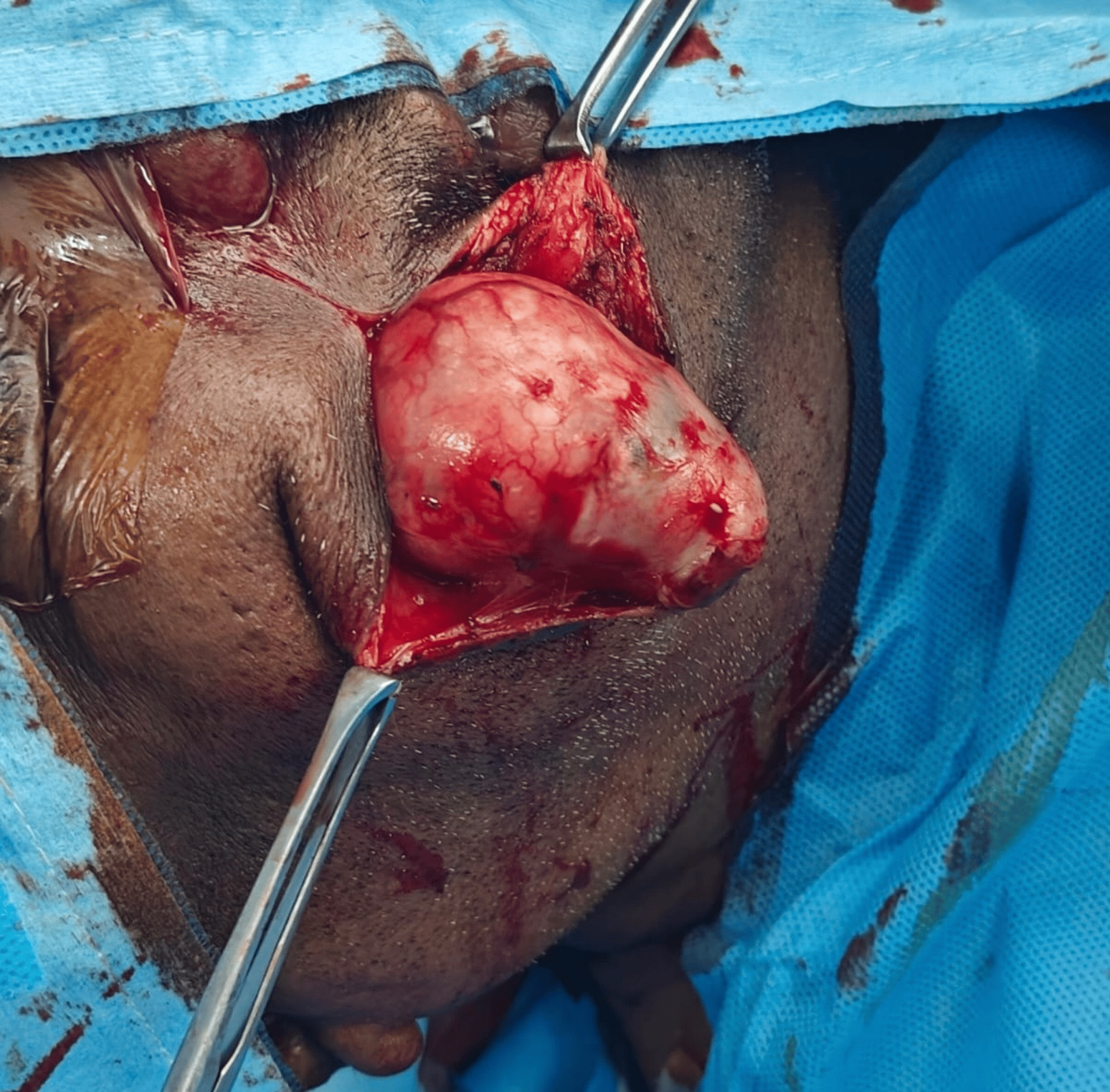
Intraoperative photograph demonstrating the use of a parotidectomy-style incision for safe identification and excision of an accessory parotid pleomorphic adenoma

**Figure 4:**
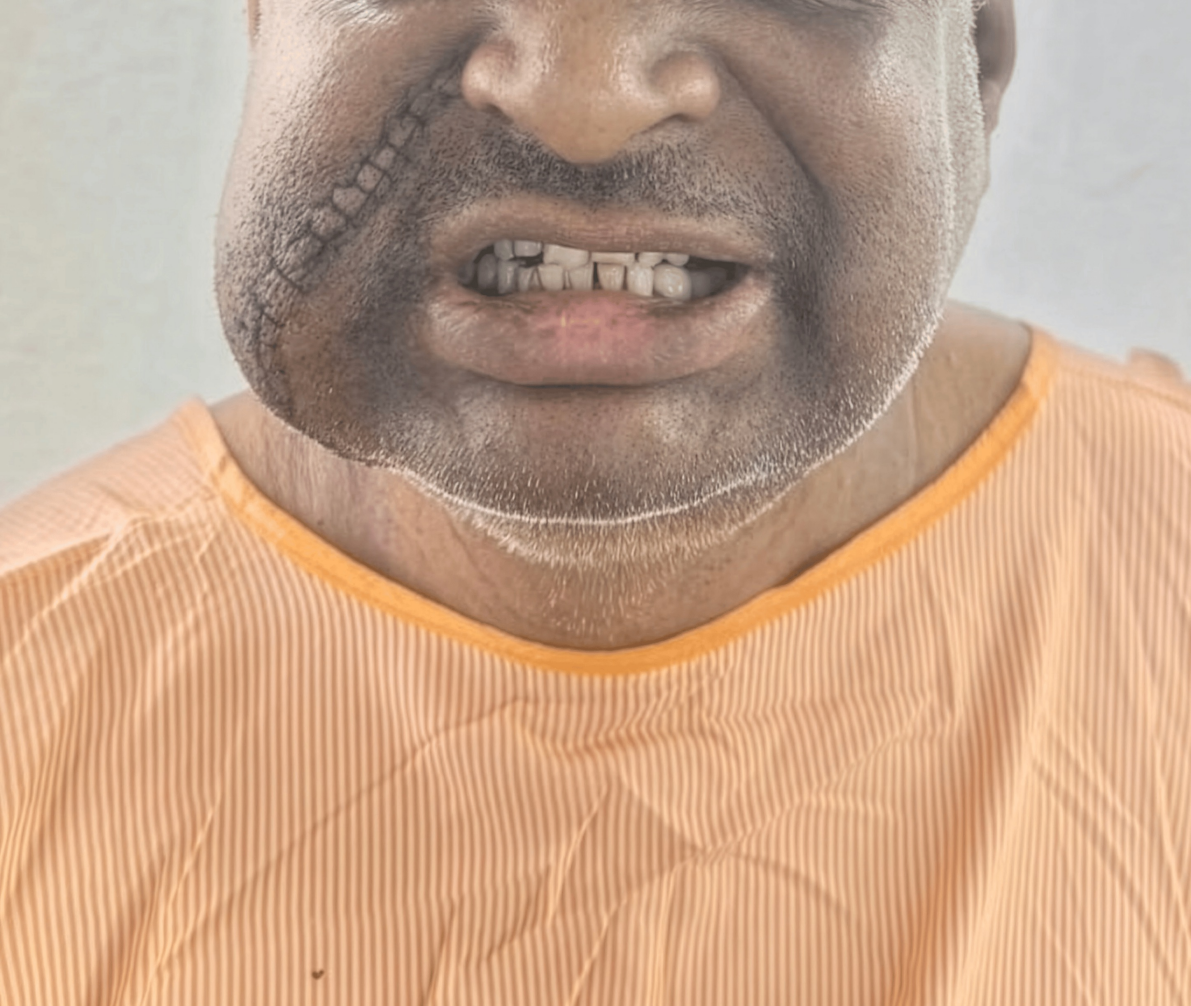
Postoperative image demonstrating satisfactory wound healing with an intact suture line and no clinical evidence of facial nerve dysfunction

Complete surgical excision of the lesion was performed under general anesthesia using a parotidectomy-type approach. This approach allowed safe identification and preservation of facial nerve branches (Figure 3). The tumor was excised in toto without spillage. The postoperative course was uneventful, with no facial nerve dysfunction (Figure 4).

Histopathology

**Figure 5:**
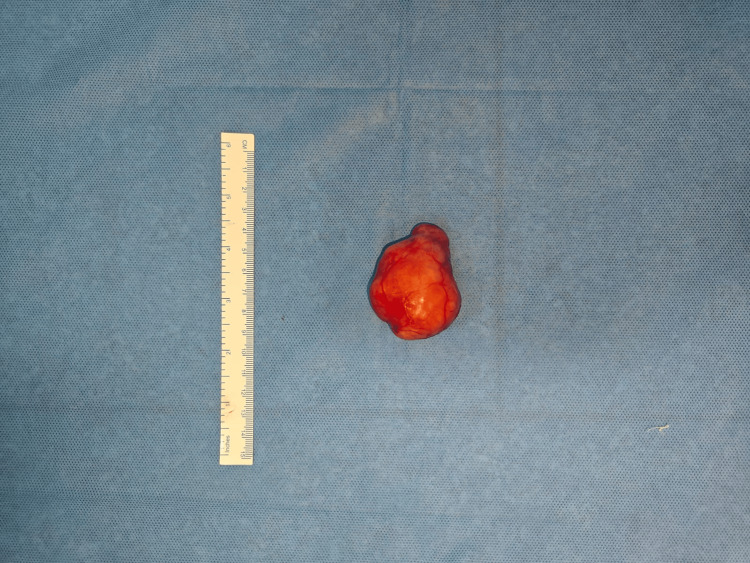
Image of the gross specimen of the accessory parotid pleomorphic adenoma

**Figure 6:**
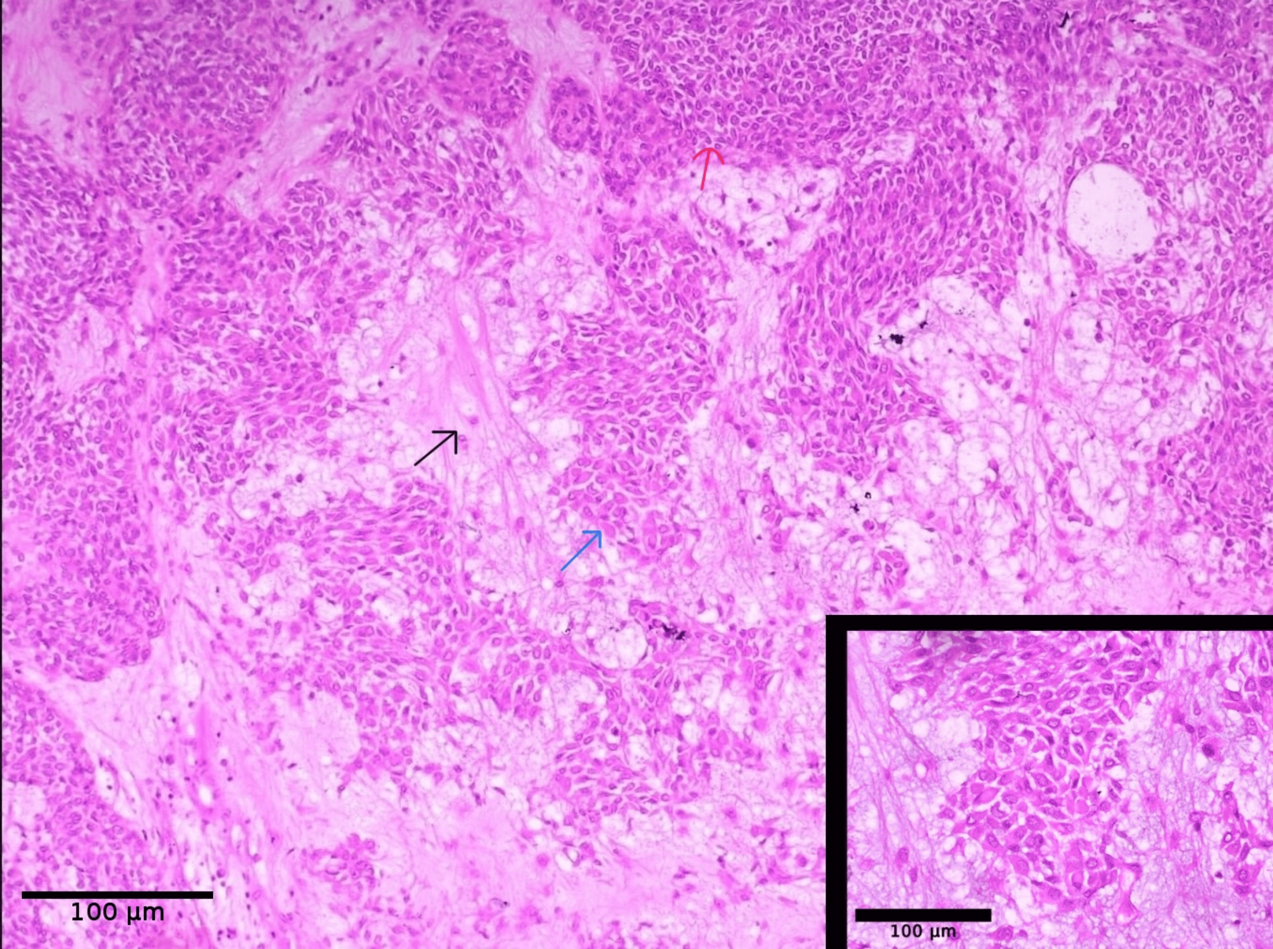
Microscopic image showing a biphasic tumor with epithelial and myoepithelial cells set in a chondromyxoid stroma background (hematoxylin and eosin stain, x 20 magnification). The bottom-right corner shows a higher magnification (x 40 magnification) of the same image. The black arrow represents a chondromyxoid stromal background, the blue arrow represents epithelial cells, and the red arrow represents myoepithelial cells.

The mass was excised and sent to histopathology for complete analysis. On gross examination, the mass was well-circumscribed, nodular on the external aspect, and measured 5.5 x 3.5 x 3.5 cm (Figure 5). The cut surface showed a solid and cystic lesion with a characteristic gelatinous appearance, seen focally. Microscopic examination of the lesion showed the presence of a biphasic tumor with epithelial and myoepithelial cells, set in a chondromyxoid stroma background. The epithelial cells were arranged in sheets, tubules, and anastomotic trabeculae closely associated with the presence of myoepithelial cells, which were spindle-shaped or plasmacytoid morphologically (Figure 6). There was no atypia, increased mitosis, or necrosis seen in the multiple sections studied. With these characteristic histologic findings, a diagnosis of pleomorphic adenoma was given. Histopathological examination also demonstrated an intact and well-defined capsule without evidence of pseudopodial extensions, and all surgical margins were free of tumor involvement.

## Discussion

Tumors of the accessory parotid gland are rare, accounting for approximately 1-7.7% of all parotid gland neoplasms [3,5]. Among these, pleomorphic adenoma represents the most common benign histological subtype, although a relatively higher proportion of accessory parotid gland tumors are reported to be malignant compared to those arising in the main parotid gland [3,6]. The accessory parotid gland is located anterior to the main parotid gland, overlying the masseter muscle and in close association with Stensen’s duct, which explains the unique clinical presentation of these lesions [1,2].

Clinically, accessory parotid tumours typically present as slow-growing, painless mid-cheek masses, often leading to diagnostic confusion with lesions of the buccal space, lipomas, epidermoid cysts, or other subcutaneous swellings [4]. Facial nerve dysfunction is uncommon at presentation due to the benign nature of pleomorphic adenomas, although the intimate relationship of the tumor with the buccal and zygomatic branches of the facial nerve has important surgical implications [4]. A high index of suspicion is therefore required when evaluating persistent mid-cheek swellings.

Imaging plays a pivotal role in diagnosis and preoperative planning. Magnetic resonance imaging (MRI) is considered the modality of choice due to its superior soft tissue resolution and ability to delineate tumor margins and define relationships with adjacent structures such as Stensen’s duct and facial nerve branches.

Fine-needle aspiration cytology (FNAC) may further aid in establishing a preoperative diagnosis, although sampling errors can occur [7]. FNAC was not performed in this case, as imaging findings were strongly suggestive of a benign, well-circumscribed accessory parotid lesion, and surgical excision was indicated irrespective of cytological confirmation. Although FNAC is a useful, minimally invasive diagnostic tool for salivary gland tumors, its role in accessory parotid lesions is debated due to the small size, variable location, and proximity to branches of the facial nerve, which may limit accuracy and carry a risk of sampling error or procedure-related complications. Furthermore, in cases with characteristic clinical and radiological features, FNAC may not significantly alter management.

Complete surgical excision remains the gold standard treatment. Various surgical approaches have been described, including direct cheek incision and standard parotidectomy-type approaches. However, a parotidectomy-type incision is widely advocated, as it allows formal identification and preservation of the facial nerve branches, thereby reducing the risk of iatrogenic nerve injury and tumor spillage. Inadequate excision or capsular rupture may predispose to recurrence, which is a known concern in pleomorphic adenoma due to its pseudopod-like extensions.

Histopathologically, pleomorphic adenoma is characterized by a mixture of epithelial and myoepithelial components within a chondromyxoid stroma, accounting for its “mixed tumour” designation. Despite its benign nature, long-standing lesions carry a risk of malignant transformation into carcinoma ex pleomorphic adenoma, further emphasising the importance of early diagnosis and complete excision [8]. Immunohistochemistry (IHC) has a supportive role in the diagnosis of pleomorphic adenoma, particularly in cases with atypical morphology or limited biopsy material. The biphasic nature of the tumor can be confirmed by demonstrating epithelial markers, such as cytokeratins and epithelial membrane antigen (EMA), along with myoepithelial markers, including S100 protein, p63, smooth muscle actin (SMA), and glial fibrillary acidic protein (GFAP), highlighting the dual cellular differentiation. However, in lesions with characteristic histomorphology, such as in the present case, IHC is not routinely required for diagnosis.

Overall, accessory parotid pleomorphic adenoma, though uncommon, should be considered in the differential diagnosis of mid-cheek masses. Accurate preoperative assessment, careful surgical planning with emphasis on facial nerve preservation, and complete excision are essential to achieve optimal outcomes and minimize recurrence.

## Conclusions

Accessory parotid pleomorphic adenoma is an uncommon but important differential diagnosis in patients presenting with mid-cheek swellings. Because of its location and overlapping clinical features, it can be easily mistaken for other lesions of the buccal space. Careful preoperative evaluation is therefore essential, with MRI being particularly useful for accurate localization and assessment of its relationship to surrounding structures. Surgical excision remains the mainstay of treatment and should be performed with attention to preserving facial nerve branches. Complete removal is important to minimize the risk of recurrence. Accurate imaging and meticulous surgical excision are key to achieving excellent functional and oncological outcomes.

## References

[1] Frommer J (1977). The human accessory parotid gland: its incidence, nature, and significance. Oral Surg Oral Med Oral Pathol.

[2] Toh H, Kodama J, Fukuda J, Rittman B, Mackenzie I (1993). Incidence and histology of human accessory parotid glands. Anat Rec.

[3] Johnson FE, Spiro RH (1979). Tumors arising in accessory parotid tissue. Am J Surg.

[4] Newberry TR, Kaufmann CR, Miller FR (2014). Review of accessory parotid gland tumors: pathologic incidence and surgical management. Am J Otolaryngol.

[5] Lin DT, Coppit GL, Burkey BB, Netterville JL (2004). Tumors of the accessory lobe of the parotid gland: a 10-year experience. Laryngoscope.

[6] Klotz DA, Coniglio JU (2000). Prudent management of the mid-cheek mass: revisiting the accessory parotid gland tumor. Laryngoscope.

[7] Zbären P, Triantafyllou A, Devaney KO, Poorten VV, Hellquist H, Rinaldo A, Ferlito A (2018). Preoperative diagnostic of parotid gland neoplasms: fine-needle aspiration cytology or core needle biopsy?. Eur Arch Otorhinolaryngol.

[8] Antony J, Gopalan V, Smith RA, Lam AK (2012). Carcinoma ex pleomorphic adenoma: a comprehensive review of clinical, pathological and molecular data. Head Neck Pathol.

